# Designing a Midwife-Led Birth Center Program Based on the MAP-IT Model: A Sequential Explanatory Mixed-Methods Study

**DOI:** 10.1186/s12978-024-01824-y

**Published:** 2024-07-04

**Authors:** Mohaddeseh Bakhshi, Sanaz Mollazadeh, Talat Khadivzadeh, Javad Moghri, Azadeh Saki, Mahboobeh Firoozi

**Affiliations:** 1 Candidate at the Department of Midwifery, Research Student Committee, Mashhad University of Medical Sciences, Mashhad, Iran; 2 Professor assistance, Nursing and Midwifery Care Research Center, Mashhad University of Medical Sciences, Mashhad, Iran; 3 Department of Management Sciences and Health Economics, School of Health Management & Social Determinants of Health Research Center, Mashhad University of Medical Sciences, Mashhad, Iran; 4 Department of Biostatistics, School of Health, Social Determinants of Health Research Center, Mashhad University of Medical Sciences, Mashhad, Iran; 5 Department of Midwifery, School of Nursing and Midwifery, Mashhad University of Medical Sciences, Mashhad, Iran

**Keywords:** Attitude, Preference, Midwifery care, Birth center, Midwifery-led care, Low-risk pregnancy, MAP-IT model

## Abstract

**Background:**

In recent decades, medical supervision of the labor and delivery process has expanded beyond its boundaries to the extent that in many settings, childbirth has become a medical event. This situation has influenced midwifery care. One of the significant barriers to midwives providing care to pregnant women is the medicalization of childbirth. So far, the policies and programs of the Ministry of Health to reduce medical interventions and cesarean section rates have not been successful. Therefore, the current study aims to be conducted with the purpose of “Designing a Midwife-Led Birth Center Program Based on the MAP-IT Model”.

**Methods/design:**

The current study is a mixed-methods sequential explanatory design by using the MAP-IT model includes 5 steps: Mobilize, Assess, Plan, Implement, and Track, providing a framework for planning and evaluating public health interventions in a community. It will be implemented in three stages: The first phase of the research will be a cross-sectional descriptive study to determine the attitudes and preferences towards establishing a midwifery-led birthing center focusing on midwives and women of childbearing age by using two researcher-made questionnaires to assess the participants’ attitudes and preferences toward establishing a midwifery-led birthing center. Subsequently, extreme cases will be selected based on the participants’ average attitude scores toward establishing a midwifery-led birthing center in the quantitative section. In the second stage of the study, qualitative in-depth interviews will be conducted with the identified extreme cases from the first quantitative phase and other stakeholders (the first and second steps of the MAP-IT model, namely identifying and forming a stakeholder coalition, and assessing community resources and real needs). In this stage, the conventional qualitative content analysis approach will be used. Subsequently, based on the quantitative and qualitative data obtained up to this stage, a midwifery-led birthing center program based on the third step of the MAP-IT model, namely Plan, will be developed and validated using the Delphi method.

**Discussion:**

This is the first study that uses a mixed-method approach for designing a midwife-led maternity care program based on the MAP-IT model. This study will fill the research gap in the field of improving midwife-led maternity care and designing a program based on the needs of a large group of pregnant mothers. We hope this program facilitates improved eligibility of midwifery to continue care to manage and improve their health easily and affordably.

**Ethical code:**

IR.MUMS.NURSE.REC. 1403. 014.

## Abstract (plain language summary)

In recent decades, medical management of the labor and delivery process has extended beyond its limitations to the extent that in many settings, childbirth has become a medical event. This situation has influenced midwifery care. The global midwifery situation indicates that one in every five women worldwide gives birth without the support of a skilled attendant. One of the significant barriers to midwives providing care to pregnant women is the medicalization of childbirth. In industrialized countries, maternal and infant mortality rates have decreased over the past 60 years due to medical or social reasons. So far, the policies and programs of the Ministry of Health to diminish medical interventions and cesarean section rates have not been successful. Midwifery models in hospital care contain midwives who support women’s choices and diverse ideas about childbirth on the one hand, and on the other hand, they must adhere to organizational guidelines as employees, primarily based on a medical and pathological approach rather than a health-oriented and midwifery perspective. Therefore, the current study aims to be conducted with the purpose of “Designing a midwifery-led birth centered maternity program based on the MAP-IT model”. It is a Model for Implementing Healthy People 2030, (Mobilize, Assess, Plan, Implement, Track), a step-by-step method for creating healthy communities. Using MAP-IT can help public health professionals and community changemakers implement a plan that is tailored to a community’s needs and assets.

## Background

The global midwifery situation in 2021 indicates that one in every five women worldwide gives birth without the support of a skilled attendant [1]. Childbirth without midwifery assistance and the increasing rates of cesarean sections hinder the achievement of Sustainable Development Goals (SDGs) 3 and 5 by 2030 and diminish maternal health indicators [2, 3]. Therefore, the World Health Organization has identified improving the quality of life for mothers and children as a key global health priority and ensuring assistance during childbirth as a guarantee for mothers and children to achieve complete health with minimal care [4]. A study conducted by UNFPA, ICM, and WHO in 88 low- and middle-income countries revealed that global access to midwifery-led care could prevent 67% of maternal deaths, 64% of infant deaths, and 65% of stillbirths, leading to saving 4.3 million lives annually by 2035 [5].

In developed countries, maternal and infant mortality rates have decreased over the past 60 years due to medical or social reasons [6–8]. However, medical supervision of labor and childbirth has extended beyond its boundaries, turning childbirth into a medical event in many settings. In this event, midwifery models in hospital care include midwives who support women’s choices and diverse ideas about childbirth on the one hand, and on the other hand, they must adhere to organizational guidelines as employees, primarily based on a medical and pathological approach rather than a health-oriented and midwifery perspective. Consequently, as the medical approach, culturally characterized by technocratic (technocratic power), hierarchical, and bureaucratic (bureaucratic rule) dimensions, stands out, midwives in hospital maternity units must base their work on conflicting care models and different belief systems, affecting midwifery care and presenting healthcare professionals with new challenges such as unnecessary medical interventions in low-risk pregnancies and physiological childbirth [9, 10]. To address and overcome this issue, many countries are implementing alternative care models that enhance maternal and infant outcomes by avoiding unnecessary medical interventions and promoting the normalcy of pregnancy and childbirth processes [6]. In countries like the United States, New Zealand, Scotland, Sweden, Iceland, and South Africa, there are midwifery care models that guide training and education, reflecting adaptability and cultural differences [11]. Some midwifery care models include Women-With-Midwife [12], Exemplary Midwifery Practice [13], Midwifery At High Risk [14], Woman-Centered SA [15], Woman-Centered Nordic [16], and Midwife-Led-Care [11, 17].

Qualitative studies have been conducted on perceptions, challenges, barriers, and executive solutions of midwifery care, and some of them are reviewed below.

The qualitative study results by Bogren et al. (2023), conducted in India with the aim of “identifying background factors influencing the implementation of midwifery care based on the process evaluation framework proposed by Moore et al.,” showed that for designing and implementing policies and interventions related to units led by midwives, factors such as providing legal frameworks enabling midwives to provide full-scope care in line with the midwifery philosophy and global standards, optimizing interdisciplinary teamwork and the knowledge and skills required for implementing the midwifery philosophy through pre-service and in-service training, accepting midwifery leadership as a key role in planning and implementing midwifery care in Midwifery-Led Care Units (MLCUs), and creating demand among women through effective messages on midwifery care and support for it should be considered [18]. A qualitative study by Malfair et al. (2015) to elucidate the perceptions of women and healthcare providers regarding establishing midwifery-led units in a university hospital in Switzerland demonstrated that the perceptions of women and healthcare providers support the implementation of midwifery-led units to promote physiological childbirth. From the perspective of women, barriers related to midwifery-led units were a lack of awareness of midwives’ scope of practice, while barriers for midwives and women’s health professionals were related to the challenge of establishing good inter professional collaboration. The study findings indicate the need for “reconceptualizing” childbirth concurrently and before establishing midwifery-led units. This is a paradigm shift that goes far beyond the hospital: a shift from a medical concept to women-centered care that empowers women [6].

In the present study, program design can specify the steps and strategies for implementing a midwifery-centered delivery center to provide care during childbirth to low-risk pregnant women before the implementation phase. This clarifies the work stages for policymakers, managers, and officials, paving the way for its implementation. Following the advancement of the program’s three steps (mobilization, assessment, planning) and its readiness, the program’s accreditation will be conducted using the Delphi technique. Hopefully, this study can be a significant step in improving the childbirth care model and enhancing maternal and infant health.

### Study aim

Designing a midwife-led birth maternity center program centered on the MAP-IT model.

### Main research question

- 1- What are the characteristics of a midwife-led birth maternity center?
- 2- How is the midwife-led maternity center program based on the MAP-IT model?

### Overall objectives of study phases (Quantitative, Qualitative, and Program Design)

- 1- Determine the attitudes and preferences of participants (Maternal health care provider—mothers) towards establishing a midwife-led maternity center (Quantitative Phase 1).
- 2- Clarify the understanding and experiences of participants regarding the establishment of a midwife-led maternity center (Qualitative Phase 2).
- 3- Design and evaluate the midwife-led maternity center program (Design and Evaluation Phase 3).

### Specific objectives of the quantitative phase

- 1- Determine the attitudes of participants (maternity stakeholders—mothers) towards establishing a midwife-led maternity center.
- 2- Identify the preferences of participants regarding the establishment of a midwife-led maternity center.

### Specific objectives of the qualitative phase

- 1- Identify and determine key stakeholders in the program and analyze them (stakeholder analysis).
- 2- Clarify the understanding and experiences of stakeholders regarding the needs, solutions, challenges, barriers, and resources available in the design of a midwife-led maternity center.

### Specific objectives of the program design phase

- 1- Design a midwife-led maternity center program based on the third step of the MAP-IT model.
- 2- Evaluate the midwife-led maternity center program using the Delphi technique.

### Application objectives

The findings of this study will lead to a deep understanding of the needs, barriers, challenges, and strategies for a midwife-led maternity center for low-risk pregnant women. It will provide a basis for designing a program that is culturally appropriate and creates a suitable platform for implementation in Iranian society. This can contribute to health policy-making and planning by the Ministry of Health to improve midwifery care, increase effective fertility rates, and enhance the health of mothers and infants.

## Methods/design

The current research is a Mixed Methods Research with an Explanatory Sequential approach that will be conducted in three stages. All research has a philosophical basis, and researchers must be aware of the assumptions they use to gain knowledge in the study. These assumptions shape the research processes and guide the study [19]. The paradigm consists of a worldview and a general perspective on the complexities of the real world [20]. In this study, the researcher will use both quantitative and qualitative methods, as well as inductive and comparative reasoning to obtain the best answer to the research question. Therefore, a pragmatism paradigm would be suitable for this type of study.

### The combined method used in the research

The explanatory method is a two-stage sequential combined study whose main purpose is to help qualitative data clarify or extend the initial quantitative results. In this research design, the researcher first collects and analyzes quantitative data. Then, qualitative data is collected and analyzed sequentially to help explain or illuminate the quantitative results from the initial stage. The qualitative stage is constructed based on the quantitative stage. The logic behind this approach is that quantitative data and its subsequent analyses provide a comprehensive understanding of the research issue. The sequential explanatory combined method includes the “results follow-up explanation model” and the “participants’ selection model”. Although both models involve an initial quantitative stage followed by a qualitative stage, they differ in the way the two stages are connected. One emphasizes a detailed examination of the results, while the other focuses on selecting participants.

The rationale for using the sequential explanatory combined method of the participant selection type:

In the present study, the sequential explanatory method of “participant selection” is used, where based on the results of the quantitative section of the study, participants for the qualitative section are selected. This method is employed when the researcher prioritizes the second qualitative stage over the initial quantitative stage and focuses on examining the qualitative aspects of a phenomenon. In the current study, in the first stage, the attitudes and preferences of participants regarding establishing a midwifery-centered delivery center in a cross-sectional descriptive study will be examined. In the second stage, based on the information obtained from the first stage, participants will be informed and selected, and then the needs, perceptions, barriers, challenges, and operational solutions of the midwifery-centered delivery center will be evaluated with these individuals using qualitative research and the first and second steps of the MAP-IT model. Finally, based on the review of relevant literature, the results of the quantitative stage, the third step of the MAP-IT model, and the country’s indigenous culture, a midwifery-centered delivery center program will be designed.

### First stage (quantitative)

In this stage, a cross-sectional descriptive study will be used to determine the average scores of the attitudes and preferences of participants regarding the establishment of a midwifery-led birth-centered delivery center.

### Research population

All midwives working in both public and private sectors specializing and women of childbearing age.

### Sample research

A total of 120 employed midwives in both public and private sectors, and 185 women of childbearing age attending healthcare centers in the city of Mashhad with specific research criteria.

### Sample size calculation and sampling method

Given that the questionnaire items on midwives’ attitudes and preferences are 20, the required sample size for factor analysis with generalizability to the population is 100 individuals. Since the minimum sample for factor analysis should exceed 100 individuals, the sample size of midwives will be 120 individuals selected through stratified sampling from four categories of employed midwives in government hospitals, private hospitals, clinics, counseling centers, and healthcare facilities.

- Employed midwives in public hospitals: 34% = 100 × 1601/550
- Employed midwives in private hospitals and charities: 31% = 100 × 1601/498
- Employed midwives in healthcare facilities: 28% = 100 × 1601/453
- Midwives with private clinics and counseling centers: 6% = 100 × 1601/100

Based on the obtained proportions and the total sample size (120 individuals), 41 individuals from government hospitals, 37 individuals from private hospitals and charities, 34 individuals from healthcare facilities, and 8 individuals from clinics and counseling centers will be randomly selected.

The questionnaire related to women of childbearing age consists of 33 items; therefore, we need a sample of 185 individuals from women of childbearing age for the required factor analysis. These samples will be collected through multi-stage cluster random sampling from healthcare centers in the city of Mashhad, where each of the five districts in Mashhad is considered a cluster, and two or three centers will be randomly selected from each cluster. The final sample size will be 305 individuals.

### Research criteria

#### Inclusion criteria

1. Willingness to participate in the research.
2. Iranian nationality.
3. Women of childbearing age between 18–35 years who have visited healthcare centers for pre-pregnancy, pregnancy, and postpartum care.
4. Midwives working in both public and private sectors with a minimum of two years of experience in maternity wards.

#### Exclusion criteria

Unwillingness to continue cooperation and incomplete questionnaire submission.

### Research environment

Includes healthcare centers, teaching hospitals, and private hospitals in the city of Mashhad. The reason for selecting these centers is the high number of visitors and cultural diversity in different regions. The selection of multiple centers and hospitals is also based on the varied experiences and perspectives of individuals regarding the establishment of a midwifery-centered maternity center.

### Data collection tools

Two researcher-made questionnaires will be used, including three sections: A: demographic characteristics, B: attitude assessment, and C: preference assessment for evaluating the attitudes and preferences of midwives and women of childbearing age towards establishing a midwifery-led birth centered maternity center.

### Tool validity and reliability

To determine the validity of the researcher-made questionnaires assessing attitudes and preferences toward establishing a midwifery-centered maternity center, both content and face validity will be utilized. In this study, the questionnaires will be prepared based on research objectives, utilizing scientific sources and existing articles, and will be reviewed by a panel of ten reproductive health experts. Content Validity Ratio (CVR) and Content Validity Index (CVI) will be calculated to assess the relevance, clarity, and simplicity of each tool item for individual items and the overall tool. The reliability of the designed questionnaire will be determined using two methods: internal consistency and stability. To assess internal consistency, the questionnaire will be completed separately by 40 individuals from the research population (20 midwives and 20 women of childbearing age), and Cronbach’s alpha coefficient will be calculated using SPSS. Questionnaire stability will be assessed through the test–retest method, calculating Spearman’s correlation coefficient. For this purpose, 20 women and 20 midwives will be selected.

### The first step in the MAP-IT model: stakeholder coalition

The initial step in the MAP-IT process is to mobilize individuals and key organizations into a coalition. Individuals who have a role in creating healthy communities and contributing to this process should be selected. There are two important ways to engage individuals in helping to solve a problem: first, they must listen to gain a better understanding of the causes of the problem, the obstacles they face in managing or preventing it, and their ideas for solving the problem. Second, they can get involved by participating in a program that empowers them to address the challenges they are facing.

#### Creating a vision

The vision should stem from the most important needs, values, and goals of the community. It should be an ideal description of the coalition for the community and reflect the goals of the coalition members. Developing an early vision allows all coalition members to feel committed to the long-term process and enables the group to move on to the next stage of the process with a common mission.

#### *Organizing a coalition

Before contacting potential stakeholders, it is important to know exactly what you want from them. Here are a few questions to consider beforehand:

#### * Potential stakeholders’ brainstorming

Who are the potential stakeholders? How do they perceive the launch of a midwifery-centered center? As much as possible, a broad group representing all individuals in the community interested in the topic or creating a healthy community should be established. In general, engaging different groups and social sectors as much as possible is an advantage. The more participation there is in planning and addressing the issue, the more ideas will emerge, and the community will have more support for these efforts. In this section, the researcher will engage midwives and women of childbearing age, healthcare managers at the national level, the population of midwives in various groups including midwifery population, midwifery scientific association, members of midwifery counseling centers, experts in women’s health and obstetrics, insurance organizations, as well as religious groups such as seminaries for women, the women’s affairs council of the province, the custodian of the Holy Shrine of Imam Reza, and others as members of the target coalition [21, 22].

### Execution method of qualitative research

Research Environment: The research environment is the field or domain of qualitative research, which is the natural environment where the phenomenon occurs. Therefore, research in the field places phenomena in their natural setting, meaning in the actual living environment under study [23]. In this study, healthcare centers, hospitals, departmental units of Mashhad University of Medical Sciences, and relevant workplace organizations form the research environment.

### Participants

In this study, key informed participants include policymakers, healthcare managers at the national level, women’s health and midwifery specialists, academic members in midwifery, midwives, and women of childbearing age, insurance organizations, and religious groups.

### Criteria for participant involvement

1. Women of childbearing age and midwives who represent the top and bottom 10% in attitude and preferences towards establishing a maternity-centered care facility will be selected as final cases in this stage [24]. Additionally, participants with differences in specific variables and those with unexpected findings will also be chosen for interviews at this stage.
2. Managers and policymakers in midwifery or individuals with experience managing units related to mothers.
3. Members of the midwifery academic faculty and women’s health and obstetrics specialists with a minimum of two years of clinical and educational experience.

#### Exclusion criterion

The lack of willingness to continue collaboration in the study.

### Sampling method

In this research, participant selection will be done through goal-oriented sampling. Purposeful sampling is a common method where participants are selected based on specific criteria related to the research question. Selecting participants rich in experience strengthens the data. Moreover, choosing individuals with different perspectives and backgrounds provides the researcher with a deeper insight into the subject and generates richer and more diverse data [20]. Various strategies with different objectives have been proposed for this type of sampling. One of the most common strategies is maximum diversity, where individuals with diverse experiences in the field under study are utilized.

### Sample size

In this study, sampling continues until reaching conceptual saturation. Saturation occurs when no new information is obtained during data analysis and coding. Sandelowski states that saturated data shows an appropriate sample size. Saturated data indicates repetition in the layers, which creates confidence and complete understanding. Sample size is discussed in qualitative research, but a number between 15 to 25 participants is likely suggested [25].

### Data collection process in qualitative study

After obtaining permission from the research deputy of Mashhad University of Medical Sciences, receiving the ethics code from the ethics committee of Mashhad University of Medical Sciences, and receiving an introduction letter to conduct research in research environments, the researcher will attend to collect data through face-to-face and virtual interviews and note-taking. One of the best and fundamental methods of collecting information is through interviews, which allows individuals to express their opinions freely about the research topic [26]. After obtaining written consent forms, the research objectives will be explained to the participants, and they will be assured that the information received will be completely confidential, and they have the right to withdraw from the study at any stage. Interviews will be conducted individually, face-to-face, and virtually. The time and place of the interview will be determined with the participants’ agreement, and permission to record the interview will be obtained. Initially, some preliminary interviews will be conducted to familiarize the researcher with potential and unforeseen issues and the formation of question combinations. After a few interviews, the questions will be reviewed and changed more systematically to obtain more information. The researcher will start the interview with general questions such as age, education level, etc., to establish communication and prepare the participants. Then, an interview guide will be used, and questions will be formed based on the interview process. Finally, exploratory questions will be asked as needed, such as “Can you give me an example?” or “What do you mean?” to obtain more information. At the end of the interviews, the researcher will ask questions such as “Is there anything else you would like to add?” to ensure that the participants have expressed all their opinions.

### Sample interview questions

1. Please discuss your understanding and experience of natural childbirth process and its care.
2. Explain your perspectives and opinions on low-risk maternal care centers.
3. Describe your understanding and experience of maternity centers managed by midwives.
4. What is your opinion on giving birth in midwifery-led birth centers (similar to home birth)?

Sample Questions for Specialized Physicians, Midwifery Managers, Midwives, Healthcare

### Personnel, policy makers, and key informants

1. What is your view on a midwifery-focused care center?
2. In your opinion, how should preferred care for low-risk mothers be?
3. What solutions do you think exist for establishing such centers?
4. What obstacles do you think exist in establishing such centers?
5. What actions can be taken to overcome these obstacles?
6. What resources (financial, human resources, physical space) do you think are needed to implement a midwifery-focused care center?

### Qualitative data analysis method

The analysis of the conducted interviews will be carried out based on the Graneheim and Lundman content analysis method (2020).

### Combining in a sequential explanatory composite design

In this study, quantitative data is first collected and analyzed, followed by connecting the quantitative findings to the qualitative phase [24]. Then, qualitative data is gathered and analyzed. Integration involves linking the quantitative results to the collection of qualitative data. The current study will utilize two methods of integrating and merging data. Integration will occur by selecting participants from the qualitative stage among those involved in the quantitative phase of the study. After analyzing the findings of the quantitative part of the study, the top 10% and bottom 10% in terms of attitude scores and preferences towards establishing a mother-centered maternity center will be identified. Participants in the second stage of the study, i.e., the qualitative phase, will be selected from among them. Integration in the synthesis phase combines the quantitative and qualitative results. This is done by comparing the results of both stages and then merging them to identify the dimensions and features for the establishment of a suitable mother-centered maternity center in the Iranian community. The data will be used to design a program based on the third step of the MAP-IT model.

### Data validity and reliability

Linken and Guba (1985) argue that maintaining the credibility of a research report depends on factors that have been discussed quantitatively as validity and reliability. The idea of discovering truth through reliable and valid measurement is replaced by the concept of “trustworthiness”, which generates confidence in the findings. These researchers proposed four criteria for ensuring the credibility of qualitative research: credibility, dependability, confirmability, and transferability [23].

### Step 2: evaluation

The next step in the MAP-IT approach is evaluating community needs and assets (resources). Needs can be defined as the gap between what is and what should be. A need can be felt by an individual, a group, or the entire community, ranging from basic needs like food and water to abstract needs like improving social cohesion. Resources or assets can include individuals, organizations and institutions, buildings, equipment—anything that can be utilized to enhance quality of life. Each individual is a potential asset to the community, and everyone possesses assets that can be used to build the community.

### Step 3 of the MAP-IT model: planning

VMOSA^1^ (Vision, Mission, Objectives, Strategies, and Action Plans) is a practical planning process used to assist social groups in defining a vision and developing practical ways to implement change. This comprehensive planning tool can help organizations move from dreams to actions and positive outcomes for the community by providing a blueprint.

The next step in the VMOSA process is developing strategies. Strategies explain how the program will achieve its objectives. Generally, organizations have a wide range of strategies that involve individuals from all sectors or different parts of the community. These strategies range from very broad strategies that encompass individuals and resources from various parts of the community to very specific ones that target precisely defined areas. Examples of broad strategies include:

- A child health program may use social marketing to promote adult participation with children.
- An adolescent pregnancy program may decide to undertake.
- The urban revitalization project may improve community artistic life by encouraging artists to perform in the area.

The current study will use the VMOSA framework to develop a program, starting with developing a vision, which involves enhancing midwifery care models and promoting natural childbirth. Subsequently, the mission, objectives, necessary strategies to achieve the objectives, and finally, the operational midwifery-centered childbirth program will be formulated. After advancing through the first three steps of the MAP-IT model, a midwifery-centered childbirth program will be designed [27].

#### Delphi technique in program on maternal care

Technique as a systematic method for extracting professional group opinions and judgments from a group of experts and independent individuals on a specific topic or question, or reaching group consensus through a series of questionnaire-driven rounds with respondent anonymity and feedback to panel members. The Delphi process involves the use of questionnaires, experts, controlled feedback, anonymity, results analysis, consensus, timing, and coordinating team. Delphi is a general term related to a set of processes used to refine the viewpoints of expert groups and qualified individuals and to strive for expert consensus on a specific issue [28–30]. The main goal of Delphi is future prediction, but it is also used in decision-making, increasing efficiency, judgment, problem-solving facilitation, needs assessment, goal setting, aiding in planning, priority setting, future prediction, creativity, group communication organization, group information gathering, respondent group training, policy determination, resource allocation, and group consensus or agreement [29–31]. Given that this study aims to present a program and make decisions based on expert consensus, a modified classic Delphi technique will be used. In this study, the Delphi process will be conducted in two or three rounds:

### First round

The initial questionnaire is sent in an unstructured or open-response format, acting as a strategy to generate ideas, to identify all relevant topics related to the study title. In this research, for the first round of Delphi, 15 stakeholders including health policymakers, healthcare managers, midwifery population, academic midwifery members, midwives, and women’s and maternal health specialists will be selected, and the Maternal-Centered Delivery Center program will be sent to them via email for them to express their opinions on the program and its components in agreement or disagreement. After collecting opinions, the responses of the participants will be analyzed and summarized by the research team.

### Second round

In the second round, questionnaires often utilize structured formats with categorized lists of activities. Similar to the first round, participants are asked to identify agreements and disagreements, providing a space for the identification of new ideas, corrections, interpretations, deletions, and explanations to strengthen and clarify their strengths and weaknesses. In some cases, participants are even requested to present arguments and reasons for prioritizing their items.

### Third round

A summary of the responses from the previous stage’s panelists will be prepared. Titles that receive less than 50% agreement will be removed. Those with agreement between 50–70% will be sent back to the panelists from the first stage. Consensus will be considered at 70% agreement and above.

## Discussion

In countries such as Northern Europe, New Zealand, and Scotland, midwifery is seen as a strong and independent profession. In the United States, midwives typically provide only a small portion of prenatal and childbirth care. All these models focus on supporting women’s independence and involving them in the care process [11, 15, 16]. This woman-centered care can be defined as a “philosophy of midwifery and a consciously chosen tool for managing care of pregnant women”, [32] which includes various care models and dimensions such as mutual respect, shared decision-making, continuity of care, relationship, and empowerment [11, 17, 33]. Among these models, only the Scandinavian Midwifery Model (MiMo) emphasizes the performance of midwives in the maternity sector, focusing on the needs and perspectives of pregnant and birthing women. Woman-centered midwifery care, both inside and outside hospitals, ensures continuity of care and is provided by one or two midwives from the beginning of pregnancy to after birth. The goal is to limit interventions and provide information that enables women to choose their care based on the assumption that pregnancy and childbirth are normal life events [34, 35]. These essential elements based on evidence are included in the path to midwifery 2030, designed to facilitate optimal midwifery performance for countries with high, medium, and low incomes, focusing on increasing community-based services and culturally appropriate care [36]. From the 1980s to the early 21st century, Australia has witnessed the evolution of midwifery-led care models. The first birthing centers in this country were introduced in the 1980s as a new model of care based on a non-interventionist philosophy, where midwives managed the centers and provided primary care. The birthing centers were mostly located within hospitals but were distinct from maternity wards [37]. Midwifery care in Australia is provided in three forms: team midwifery, private practice midwifery (PPM), and group midwifery practice (MGP) [38]. During the 1960s and 70s, alternative birthing methods were introduced in Canadian midwifery society [39]. In July 2018, the first Midwifery Unit (AMU) was opened at Markham Stouffville Hospital (MSH) with distinct policies and protocols from traditional maternity units. Primary care during childbirth is provided by community-based midwives working in a continuous care model. This unit is managed by a hospital midwife who provides clinical support and guidance to community midwives. In addition to management, the hospital midwife facilitates admissions and discharges, liaises with consultants, supports learners, and acts as a second midwife during childbirth [8]. In the United States, the term “Birth Center” encompasses organizational models including locations led by midwives or jointly managed by midwives and obstetricians. Research has been conducted since 1990 to expand independent birthing units, resulting in 37 states currently having birth centers [40]. In the United States, Certified Nurse-Midwives (CNMs), Certified Midwives (CNs), and Certified Professional Midwives (CPMs) are service providers in midwifery-centered birthing centers. These centers follow midwifery principles focusing on pregnancy through a lens of health, emphasizing optimizing natural physiological childbirth and providing continuous support with interventions only when necessary. Core principles include respecting cultural differences, supporting maternal autonomy, and limiting interventions unless medically necessary. Midwifery-centered care processes are distinctly different from traditional hospital-based birth center models. Prenatal visits are three to four times longer, emphasizing education, empowerment, and shared decision-making. Additionally, in these centers, childbirth occurs in a non-medical environment that encourages freedom of movement, self-directed nutrition, and active involvement of support individuals. Common care processes such as intermittent fetal heart rate monitoring and vital sign checks are present, but medical interventions like continuous external fetal monitoring, intravenous fluids, and constant monitoring of maternal vital signs are not performed [41]. The current definition of the International Confederation of Midwives (ICM) of the Midwifery-Led Birth Center (MLBC) is a healthcare facility that provides maternity, sexual, and reproductive health services using a midwifery care model. Midwives are responsible for low-risk childbirth care, ensuring access to emergency care, and are fully integrated into the healthcare system. A midwifery center stands out due to its alignment with the philosophy of midwifery care. This human rights and women-centered approach, expressed through a shared home-like space that encourages women’s and community participation, sets it apart. The midwifery center aligns the level of care provided with evolving needs to deliver optimal outcomes. Care provided at a midwifery center is oriented towards guiding and directing the woman’s experience [7]. Another broader and as comprehensive as possible definition includes: “A dedicated space for childbirth care providers, where midwives take primary professional responsibility for childbirth care”. This general definition encompasses various types of midwifery-led care centers, including independentx`, alongside, and on-site (located within the hospital’s maternity unit) [42]. We hope the present study, and program design can specify the steps and strategies for implementing a midwifery-centered delivery center to provide care during childbirth to low-risk pregnant women before the implementation phase.

## Data Availability

No datasets were generated or analysed during the current study.

## Abbreviations

MAP-IT Model: Mobilize, Assess, Plan, Implement, Track
VMOSA: Vision, Mission, Objectives, Strategies, and Action Plans

## Footnotes

1 **V**ision, **M**ission, **O**bjectives, **S**trategies, and **A**ction Plans.

## References

[1] International Confederation of Midwives (ICM). Position Statement. Midwives’.Midwifery Led Care, The First Choice For All Women. 2019.32993311

[2] Sangy M T, Duaso M, Feeley C , Walker Sh. Barriers and facilitators to the implementation of midwife-led care for childbearing women in low- and middle-income countries: A mixed-methods systematic review. Midwifery. 2023;122:103696. 10.1016/j.midw.2023.103696 37099826

[3] Franchi JVO, Pelloso SM, Ferrari RAP, Cardelli AAM. Access to care during labor and delivery and safety to maternal health. Rev Lat Am Enfermagem. 2020;28:e3292. 32520244 10.1590/1518-8345.3470.3292 PMC7282715

[4] WHO Recommendations on antenatalcare for a positive pregnancy experience. WHO Library Cataloguing. 2016:176.

[5] Nove A, Friberg IK, de Bernis L, Mc Conville F, Moran AC, Najjemba M, et al. Potential impact of midwives in preventing and reducing maternal and neonatal mortality and stillbirths: a lives saved tool modelling study. The Lancet Global Health. 2021;9(1):e24–32. 33275948 10.1016/S2214-109X(20)30397-1 PMC7758876

[6] Maillefer F, Labrusse C, Cardia-Vonèche L, Hohlfeld P, Stoll B. Women and healthcare providers’ perceptions of a midwife-led unit in a Swiss university hospital: a qualitative study. BMC Pregnancy Childbirth. 2015;11(15):56. 25886389 10.1186/s12884-015-0477-4 PMC4359486

[7] Stevens JR , Alonso C. Commentary: creating a definition for global midwifery centers. Midwifery. 2020;85:102684. 32182449 10.1016/j.midw.2020.102684

[8] Darling EK, Easterbrook R, Grenier LN , Malott A , Murray-Davis B, Mattison CA. Lessons learned from the implementation of Canada’s first alongside midwifery unit: A qualitative explanatory study. Midwifery. 2021;103:103146. 34592575 10.1016/j.midw.2021.103146

[9] Hunter B. Conflicting ideologies as a source of emotion work in midwifery. Midwifery. 2014;20(3):261–72. 15337282 10.1016/j.midw.2003.12.004

[10] Blaaka G, Schauer Er T. Doing midwifery between different belief systems. Midwifery. 2008;24(3):344–52. 17316937 10.1016/j.midw.2006.10.005

[11] Berg M, Asta Ólafsdóttir Ó, Lundgren I. A midwifery model of woman-centred childbirth care In Swedish and Icelandic settings. Sex Reprod Healthc. 2012;3(2):79–87. 22578755 10.1016/j.srhc.2012.03.001

[12] Fleming V. Women-with-midwives: a model of interdependence. Midwifery. 1998;14(3):137–43. 9856020 10.1016/s0266-6138(98)90028-6

[13] Kennedy HP. A model of exemplary midwifery practice: results of a delphi study. J Midwifery Womens Health. 2000;45(1):4–19. 10772731 10.1016/s1526-9523(99)00018-5

[14] Berg M. A midwifery model of care for childbearing women at high risk: genuine caring in caring for the genuine. J Perinat Educ. 2005;14(1):9–21. 17273417 10.1624/105812405X23577 PMC1595225

[15] Maputle MS. A woman-centred childbirth model. Health SA Gesondheid. 2010;15(1):28–35. 16109595 10.1080/09513590500107660

[16] Halldorsdottir S, Karlsdottir SI. The primacy of the good midwife in midwifery services: an evolving theory of professionalism in midwifery. Scand J Caring Sci. 2011;25(4):806–17. 10.1111/j.1471-6712.2011.00886.x 21480938

[17] Lundgrena I, Berga M, Nilssonb Ch, Olafsdottirc OA. Health professionals’ perceptions of a midwifery model of woman-centred care implemented on a hospital labour ward. Women and Birth. 2020;33(1):60–9. 10.1016/j.wombi.2019.01.004 30686654

[18] Bogren M , Jha P, Sharma B, Erlandsson K. Contextual factors influencing the implementation of midwifery-led care units in India. Women Birth. 2023;36 (1):e134–41. 10.1016/j.wombi.2022.05.006 35641395

[19] Bahramnezhad F, Shiri M, Asgari P, Farokhnezhad Afshar P. A Review of the Nursing Paradigm. Open J Nurs. 2015;5(1):17–23. 30611257 10.1186/s12905-018-0705-y PMC6321671

[20] Polit DF, Beck CT. Essentials of nursing research: Appraising evidence for nursing practice. Philadelphia: Lippincott Williams & Wilkins; 2020.

[21] NRSG 780 - HEALTH PROMOTION AND POPULATION HEALTH Module 8: program planning essentials and models. Available from: https://ctb.ku.edu/en/table-of-contents/overview/modelsfor-community-health-and-development/map-it/main.

[22] Section 14. MAP-IT: A Model for Implementing Healthy People 2020. Available from: https://ctb.ku.edu/en/table-of-contents/overview/models-for-community-health-anddevelopment/map-it/main.

[23] Polit DF, Beck CT. Research manual for nursing research. Generafing and assessing evidence for nursing practice. Philadelphia: Lippincott Williams & Wilkins; 2012.

[24] Creswell JW, Plano Clark VL. Designing and conducting mixed methods research. Thousand Oaks: SAGE Publications; 2017.

[25] Drisko JW, Maschi T. Content analysis: Pocket guidesto social work research methods. Oxford: Oxford University Press; 2016.

[26] Attarha M, Keshavarz Z. The midwives, experience about midwife-mother relationship delivery room. The J Urimia Nurs Midwifery Fac. 2017;14(10):858–47. ( Persian). 26.

[27] Section 1. An overview of strategic planning or "VMOSA" (Vision, Mission, Objectives, Strategies, and Action Plans). Available from: https://ctb.ku.edu/en/table-ofcontents/structure/strategic-planning/vmosa/main.

[28] Jamali E, Habibi M, R. BY. Application of Delphi method in the behavioral sciences and medical research: a review of advantages,limitations and methodology. Higher Educ Letter. 2014;7(26):131–54. (Persian).

[29] Ahmadi F, Nasiriani K, Abazari p. Delphi technique: instrument for research. Educ Med Sci. 2008;8(1):175–85.

[30] Williams PL, Webb C. The Delphi technique: a methodological discussion. J Adv Nurs 1994;19(1):180–6. 10.1111/j.1365-2648.1994.tb01066.x 8138622

[31] Niederberger M, Spranger J. Delphi technique in health sciences: a map. Front Public Health. 2020;8:457. 10.3389/fpubh.2020.00457 PMC7536299 33072683

[32] Fontein-Kuipers Y, de Groot R, van Staa A. Woman-centered care 2.0: bringing the concept into focus. Eur J Midwifery. 2018;2:5. 10.18332/ejm/91492 PMC7846029 33537566

[33] Eri TS, Berg M, Dahl B, Gottfreðsdóttir H, Sommerseth E, Prinds Ch. Models for midwifery care: A mapping review. Eur J Midwifery. 2020;4:30. 10.18332/ejm/124110 PMC7839165 33537631

[34] WHO. Midwife-led versus other models of care for childbearing women 2016 Available from: http://apps.who.int/rhl/pregnancy_childbirth/antenatal_care/general/cd004667_Wiysongecs_com/en. 10.1002/14651858.CD004667.pub2 18843666

[35] Wallace J. Using a birth center model of care to improve peproductive outcomes in informal settlements—a case study. J Urban Health. 2019;96(2):208–18. 10.1007/s11524-018-0257-3 PMC6458220 29869316

[36] ten-Hoope-Bender P, Lopes ST, Nove A, Michel-Schuldt M, Moyo MT, Bokosi M. Midwifery 2030: a woman’s pathway to health. What does it mean? Midwifery. 2016;32:1–6. 10.1016/j.midw.2015.10.014 26621374

[37] McKellara L, Newnhamb E, Fleeta JA, Adelson P. Midwifery-led care in South Australia: Looking back to move forward. Women and Birth. 2021;34(5):e537–45. 10.1016/j.wombi.2020.10.011 33168494

[38] Hewitt L, Dahlen HG, Hartz DL, Dadich A. Leadership and management in midwifery-led continuity of care models: A thematic and lexical analysis of a scoping review. Midwifery. 2021;98:102986. 10.1016/j.midw.2021.102986 33774389

[39] Burton N, Ariss R. The critical social voice of midwifery: midwives in Ontario. Revue Canadienne de la Rechercheet de laPratiqueSage-femme 2009;8(1):7–22.

[40] Walsh D , Downe SM. Outcomes of free-standing, midwife-led birth centers: a structured review. Birth. 2004;31(3):222–9. 10.1111/j.0730-7659.2004.00309.x 15330886

[41] Wallace A, Hoehn-Velasco L, Tilden E, Dowd BE, Calvin S, Jolles DR, et al. An alternative model of maternity care for low-risk birth: Maternal and neonatal outcomes utilizing the midwifery-based birth center model. Health Serv Res. 2023;59:1–13. 10.1111/1475-6773.14222 PMC10771911 37691323

[42] Nove A , Bazirete O , Hughes K , Turkmani S , Callander E , Scarf V , et al. Which low- and middle-income countries have midwife-led birthing centres and what are the main characteristics of these centres? A scoping review and scoping survey. Midwifery. 2023;123:103717. 10.1016/j.midw.2023.103717 PMC10281083 37182478

